# Advances in rice seedling cultivation techniques in China: a comprehensive review

**DOI:** 10.3389/fpls.2025.1679753

**Published:** 2026-01-05

**Authors:** Xiaolian Yang, Zeyu Yang, Chuan Zhao, Guoliang Li, Jiangtao Li

**Affiliations:** 1 Huzhou Key Laboratory of Innovation and Application of Agricultural Germplasm Resources, Huzhou Academy of Agricultural Sciences, Huzhou, China; 2 Huzhou Key Laboratory of Intelligent Sensing and Optimal Control for Industrial Systems, School of Engineering, Huzhou University, Huzhou, China; 3 Nanchong Animal Husbandry Station of Sichuan Province, Nanchong, China

**Keywords:** greenhouse intelligent seedling raising, mechanized rice production, paddy seedling raising, rice industrialized seedling cultivation, rice seedling raising techniques

## Abstract

Rice is crucial for China’s food security. Mechanized rice production offers advantages like labor-cost reduction, efficiency gains, and yield stability. In mechanized rice production, mechanized and high-efficiency seedling raising has consistently been a top priority, with three main methods: traditional paddy nurseries, greenhouse systems, and industrialized factory production. Factory-based systems use advanced tech, enabling full-process mechanization and intelligent monitoring, reducing seedling mortality. This review explores the development trends of rice seedling raising technology focusing on rice mechanical transplanting, as well as the innovation paths for future equipment.

## 1 Introduction

As a staple food crop in China, rice plays a pivotal role in sustaining national food security. With the aging agricultural workforce and the industry’s shift toward standardized large-scale operations, mechanized rice production has demonstrated significant advantages. These include labor cost reduction (30-50% savings), 20-35% efficiency gains in planting operations, enhanced resilience against climatic extremes (Sharma, 1995), and stabilized yield outputs critical for food security maintenance (Chen et al., 2018; Lin et al., 2015).

**Figure 1:**
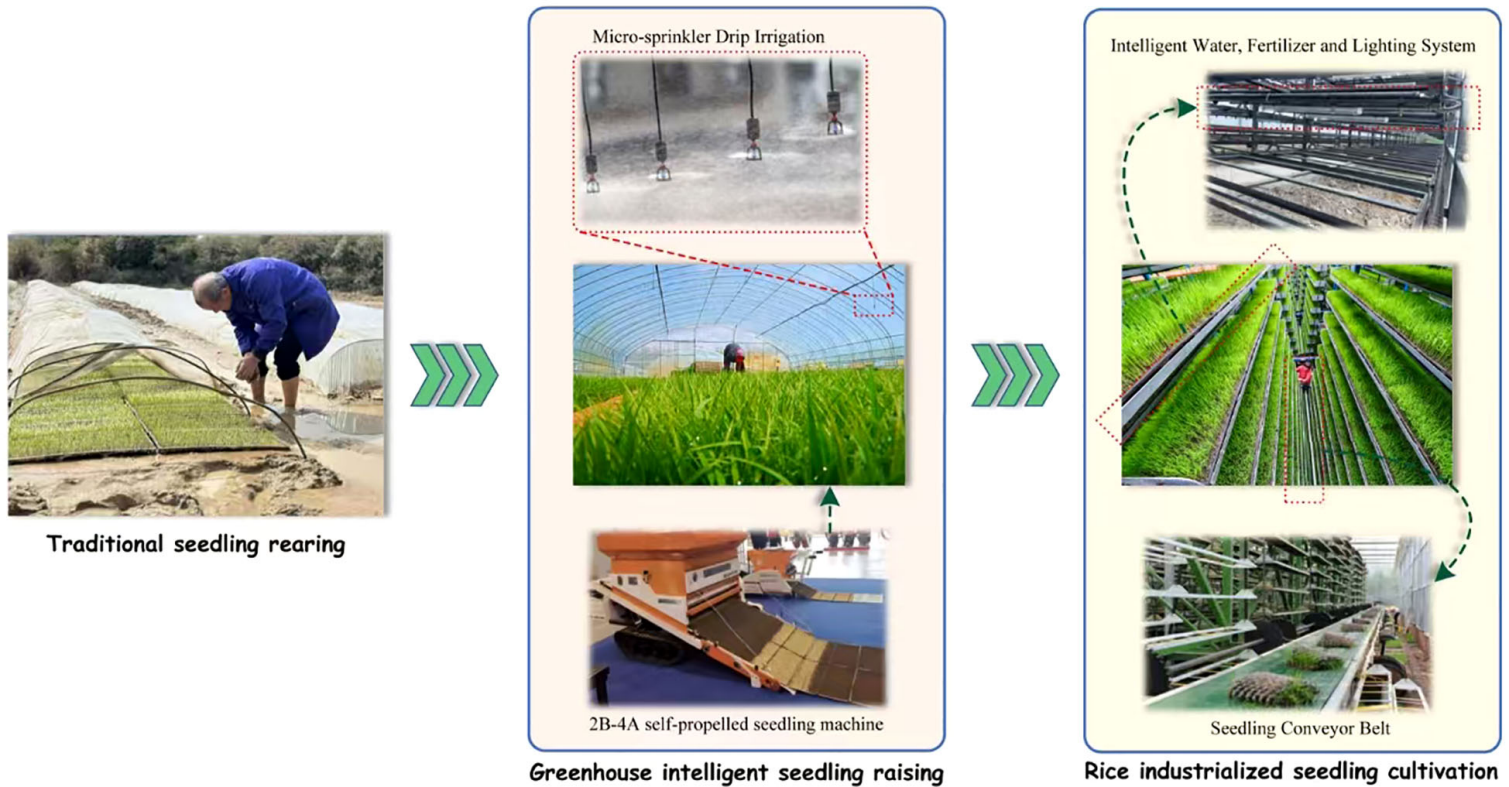
Evolution of rice seedling.

Within mechanized rice production systems, seedling cultivation emerges as the most technologically demanding phase. Current methodologies encompass three primary approaches: traditional paddy field nurseries, greenhouse-based systems, and industrialized factory production (**Figure 1**). The latter integrates advanced technologies such as multi-tier fixed-bed vertical cultivation units and rotary seedling platforms Pan and Wu, 2008; Zha et al., 2020). Factory-based systems revolutionize traditional decentralized practices through centralized environmental control, achieving full-process mechanization from precision sowing (error rate <3%) to germination chamber management (temperature control ±0.5°C). Intelligent monitoring systems regulate temperature (20-32°C), humidity (70-85% RH), and photosynthetic active radiation (PAR 400-700 μmol/m²/s), mitigating seasonal variability risks that previously caused 15-20% seedling mortality in conventional systems (Ministry of Agriculture and Rural Affairs, General Office, 2023; Yang and Jiang, 2022). This review examines the evolution of seedling cultivation technologies and proposes innovation pathways for next-generation mechanized seedling equipment.

## 2 Global and domestic research progress in rice seedling raising techniques

In the current research landscape, the methods of rice seedling nurturing both domestically and internationally vary significantly. In Western countries, such as those in Europe (Svecnjak, 2021; Busetto et al., 2017; Ferrero and Tinarelli, 2007) and America (Daum, 2023; Farooq et al., 2011), rice cultivation primarily employs direct seeding, with little to no transplanting of seedlings, hence the development of seedling nurturing Zmachinery is minimal. In contrast, Asian countries (Alimova et al., 2024) like Japan (Oshiro, 2016) and South Korea (Timsina and Connor, 2001; Lee et al., 2024; Kim, S. Y., et al., 2013) predominantly utilize factory-based seedling nurturing. The processes of seed treatment and seeding for seedlings have been fully refined. Following sowing, seedling trays are systematically placed in controlled dark (Benke and Tomkins, 2017; Lee et al., 2013) environments to initiate germination. Subsequently, germinated trays are transferred to greenhouse facilities for vegetative growth under regulated climatic conditions (Zhao et al., 2016). Cultivation occurs either on ground-level beds or multi-tiered shelving systems to optimize space utilization (Lu, 2023). As seedlings approach the late vegetative growth stage (Bochtis et al., 2014), they undergo a hardening-off process through relocation to open-field environments. While manual trolleys or carts remain the primary mode for short-distance tray transportation, recent technological integration has introduced semi-automated conveyor systems for long-distance inter-facility logistics (Linganagouda et al., 2016). However, critical operational phases-including tray loading/unloading onto conveyor belts, intermodal transfers between greenhouses and fields, and precision alignment during transplantation-still rely heavily on human intervention (Khadatkar et al., 2021). This persistent dependency on manual labor underscores the limited mechanization (Ly, 2021) penetration in seedling nurturing workflows, particularly in material handling and transitional operations, which constrains scalability and operational efficiency in large-scale production systems.

In China, mechanized rice seedling cultivation predominantly utilizes tray-based methods (Wang et al., 2018), categorized into factory-based systems and field-based approaches. Regional implementation patterns (Wang et al., 2025) show distinct geographical preferences: northern China primarily employs controlled-environment factory cultivation with specialized equipment, while southern regions adopt a hybrid model incorporating both factory and field-based systems. Although traditional field cultivation maintains relevance due to its simplified equipment requirements and reduced tray transportation logistics, it faces critical limitations including excessive land consumption, climate dependency (Feng et al., 2024), and reliance on manual irrigation/fertilization practices. These constraints result in inconsistent seedling quality and incompatibility with standardized mechanized transplanting protocols.

Conversely, factory-based cultivation (Ling et al., 2025; Zhang et al., 2024) represents an advanced agricultural technology that optimizes growth parameters through artificial climate control. This method enables mass production of uniformly high-quality seedlings, ensuring compatibility with mechanized transplanting systems while enhancing rice yield and grain quality. These operational advantages have driven its widespread adoption in modern Chinese rice cultivation practices.

## 3 Research advances in rice seedling raising techniques

### 3.1 Methods of traditional seedling rearing

Traditional rice seedling cultivation predominantly involves paddy field-based methods, characterized by open-field operations where germinated seeds develop into transplant-ready seedlings. This conventional approach, while historically prevalent, demonstrates significant vulnerability to meteorological variability and environmental fluctuations. Particularly during early-stage cultivation, unpredictable climatic shifts frequently induce physiological disorders such as seedling rot disease (Yoshimoto et al., 2011). The inherent limitations of field-dependent microclimate regulation have progressively rendered this method obsolete with advancements in protected cultivation technologies. Contemporary agricultural practices increasingly employ greenhouse systems to optimize environmental parameters, demonstrating enhanced seedling survival rates under extreme weather conditions including prolonged rainfall and temperature anomalies (Xiong et al., 2025).

### 3.2 Methods of greenhouse rice seedling raising techniques

The emergence of rice greenhouse seedling cultivation is closely associated with the development of mechanized rice transplanting (Ling et al., 2025). This technology creates an artificially controlled environment optimized for seedling growth. In recent years, rice greenhouse seedling cultivation (Gupta et al., 2010; Kim et al., 2013; Feng and Hu, 2021; Edwardson, 2013) has developed rapidly, demonstrating significant technical advantages in production including convenient operational management, low seedling-raising costs, and superior seedling quality.

This technology integrates agricultural machinery (e.g., seedling sowing machines), greenhouse facilities (e.g., drip irrigation systems (Qu et al., 2019; Arik and Korkut, 2022), side-rolling membrane windows, and sunshade (Zhang et al., 2013) nets, and agronomic practices within modern multi-span greenhouses. The technical advantages are threefold:

1. Resource efficiency and shortened seedling age: A 960 m² multi-span greenhouse can produce 12,000 seedling trays, sufficient for transplanting 40 hectares of farmland, equivalent to 0.4 hectares of open-field seedling beds, while reducing irrigation water usage. The controlled environment (temperature, humidity, etc.) shortens seedling age by 2–3 days (Zhou et al., 2018).
2. Enhanced disaster resilience: Greenhouse cultivation mitigates weather-related risks, ensuring consistent seedling quality and avoiding delays during rainy conditions (Feng et al., 2015).
3. Improved transplanting quality: Mechanized sowing ensures uniformity, high germination rates, and robust seedlings, reducing gaps during transplanting and accelerating post-transplant recovery (Xu et al., 2009; Li et al., 2015). The use of commercial substrates minimizes soil extraction.

A limitation is the labor-intensive handling of seedling trays. To address this, Jin et al. (2015) developed a self-propelled seedling machine (2B-4A model) with a crawler system, capable of automated seeding, soil covering, and tray placement. Trials demonstrated an operational efficiency of 800-2,500 trays/hour under parameters of 56 cm × 28 cm tray size, 0.12–0.4 m/s speed, and 4-row tray placement. However, conventional greenhouse structures exhibit insufficient environmental modulation capabilities, necessitating the integration of intelligent management systems incorporating real-time monitoring and automated control mechanisms (Bai et al., 2025).

### 3.3 Methods of rice industrialized seedling cultivation

Industrialized rice seedling cultivation (Ge et al., 2021) primarily comprises a seeding assembly line, automatic tray stacking system, mobile circulating seedling beds, and intelligent seedling cultivation centers. The seeding assembly line integrates seed pretreatment equipment, automated sowing devices, and seedling-raising facilities (Xu et al., 2022). In China, seed pretreatment and seeding assembly line technologies are highly mature, with standardized equipment widely adopted nationwide (Xu et al., 2022). However, the development of intelligent factory-based systems (Yang and Liu, 2024; Gui, 2024) remains uneven due to inconsistent technical standards and operational expertise. The advantages (Yang et al., 2023; Li, 2021) of factory-based seedling nurturing include efficient space utilization and the ability to artificially regulate nurturing conditions, ensuring the production of superior and consistent seedlings. A notable disadvantage, however, is the substantial financial investment required for equipment and the necessity for a suitable location to house these facilities (Ji, 2020; Hu et al., 2025).

#### 3.3.1 Multi-layer fixed-bed vertical seedling cultivation

To enhance land and space utilization in seedling cultivation while reducing seedling transportation, domestic researchers have developed three-dimensional seedling raising equipment in recent years. The simplified “丰”-shaped frame seedling technology (Li et al., 2010) in greenhouses partially improves light and heat exposure for seedlings but reduces effective utilization of seedling area. Promoting this technology shifts traditional decentralized field seedling raising to centralized greenhouse systems, where a 667 m² greenhouse can cultivate seedlings for transplanting 27.3 hm² of farmland. This approach facilitates management, saves land and labor, reduces costs, and enhances resistance to pests and natural disasters.

Multi-layer three-dimensional cultivation can save 3–5 times (Lin et al., 2016; Ma et al., 2015) the land area and has become a global research focus. However, traditional rectangular-frame three-dimensional seedling racks stack trays densely, hindering air circulation, temperature/humidity regulation, and light distribution. These limitations cause uneven growth between upper and lower layers, lowering standardization and necessitating manual tray rotation, which increases labor intensity (Gonzalez-Fuentes et al., 2016; Wang et al., 2017).

Li Qiang (Li et al., 2021) et al. designed a closed three-dimensional rice seedling system mimicking vegetable production, featuring fully intelligent control of artificial lighting, water-fertilizer supply, and greenhouse conditions. However, such high-cost vegetable-oriented systems are unsuitable for large-scale rice factory seedling production.

#### 3.3.2 Rotating seedling cultivation equipment

To improve the structure of seedling racks for enhanced cultivation quality, Gao Lihong (Gao et al., 2015) invented a multi-layer seedling rack group that utilizes the self-weight of trays to achieve automatic flipping. This design partially mitigates the impact of uneven light distribution on seedling quality, but it demands highly complex transmission systems and is prone to tray flipping incidents. Li Jingzhu (Li et al., 2015) developed a three-dimensional movable seedling rack capable of horizontal rotation around a central axis, improving light utilization. While the height between trays is adjustable, light blockage along the central axis persists during high solar elevation angles. Dong Xuecheng (Dong et al., 2015) proposed a rotating seedling rack with an overly intricate structure, complicating maintenance and tray position adjustments. The fixed relative positions between trays further hinder flexible loading and unloading operations. Liang Qi (Liang et al., 2019) designed a roller-type three-dimensional rotating seedling rack that maintains trays parallel to the ground during rotation, ensuring balanced utilization of light, temperature, and air resources. However, limited tray capacity and challenges in controlling long-distance rotating transmission systems restrict its scalability. Current three-dimensional rotating rice seedling devices rely heavily on manual labor or makeshift machinery for seedling input/output operations, resulting in low efficiency and high labor intensity. These limitations render them unsuitable for large-scale factory production, underscoring the need for automated solutions to enhance mechanization in seedling handling.

## 4 Adoption and application rates of major seedling cultivation technologies in China

From an overall development trend perspective, China’s rice seedling cultivation technology is rapidly advancing towards mechanization, intelligence, ecological sustainability, and standardization. Various seedling cultivation technologies are transitioning from the application of single techniques to the integration of comprehensive systems. Through intelligent environmental control and precision management, standardized production, intensive operation, and highly efficient utilization of rice seedling cultivation can be achieved. The major rice seedling raising technologies in China each have distinctive features, making them suitable for different ecological regions and production conditions. **Table 1** provides a comprehensive comparison of the advantages, disadvantages, and promotion status of these four primary technologies.

**Table 1:** Comparative analysis of major rice seedling raising technologies in China.

Technology type	Advantages	Disadvantages/challenges	Promotion scope	Application rate
Mechanized Seedling Raising & Transplanting(tray-grown/plug seedlings)	High operational efficiency; ~20% yield increase; Strong stress resistance; Reduces herbicide usage.	High initial investment; High technical threshold; Requires meticulous management.	Nationwide, with a focus on double-cropping rice regions in South China.	The comprehensive mechanization rate for rice cultivation exceeds 88%; considered a mainstream technology.
Greenhouse Substrate Seedling Raising	High seedling uniformity (up to98%); 3x increase in sowing efficiency; Cost savings of 120 RMB/hm^2^; Eco-friendly.	High initial construction cost; Complex technical process; Low production flexibility.	Large-scale application in Tianjin; demonstration bases in Heilongjiang and Jiangsu.	Large-scale use in Tianjin; currently at the demonstration and promotion stage nationwide.
Precision Strip-Sowing Seedling Raising	Excellent seedling quality; Significant yield increase (24%); High level of technical integration; Notable economic benefits.	High technical threshold; Requires precise management; Needs supporting technologies for best results.	Core demonstration areas in 12 pilot counties, including Guangde City, Anhui Province.	Regional demonstration stage; the technical model is ready for replication and wider promotion.

## 5 Issues and future trends in development

As the country with the largest rice-planting area globally, China features significant ecological regional disparities and rich variety diversity. This necessitates that the research and development of rice seedling-raising machinery adhere to the principle of “adapting machinery to local conditions”. Over the past four decades of technological evolution, domestic rice seedling-raising equipment has achieved a leap -forward development from introduction and digestion to independent innovation. The seedling-raising (Alimova et al., 2024; Hossen et al., 2022; Narbaria et al., 2015) and sowing assembly lines have made phased breakthroughs in integration and automation. However, when benchmarked against international advanced levels, China still lags significantly in core technology areas (Shen et al., 2010; Zheng, 2015; Wang, 2025) such as equipment reliability (the average trouble-free time is only 65% of the international standard), sowing accuracy (the qualification rate is 15–20 percentage points lower than that of developed countries), and intelligent control. With the acceleration of agricultural modernization, the market demand for intelligent seedling-raising equipment equipped with high-precision seed metering mechanisms (sowing error of ±3%), intelligent environmental regulation (temperature and humidity control accuracy of ± 1°C/± 5%RH), and Internet-of-Things remote monitoring functions is growing exponentially.

### 5.1 Analysis of existing problems

1. Seedling Technology - Bottlenecks and Intelligent Transformation: The industrialized seedling-raising system suffers from significant process disconnections, particularly the mechanization gap between sowing assembly lines and field management. The transfer of seedling trays remains a critical bottleneck restricting full-process mechanization. Equipment demonstrates insufficient adaptability to varied terrains (paddy/dry fields) and substrates (coconut coir/straw), resulting in utilization rates below 60%.
2. Economic Benefits - Cost Paradox and Innovative Models: The high initial investment required for industrialized seedling systems creates a significant barrier to entry. The seasonal nature of rice production leads to prolonged equipment idle periods, resulting in low annual asset utilization. This is exacerbated by the 5–8 year cost-recovery period for high-end equipment, which conflicts with China’s dominant small-scale farming model.
3. Environmental Impact - Resource Consumption and Sustainable Solutions: The system’s heavy reliance on electricity for climate control and automated operations increases its carbon footprint compared to traditional methods. Extensive use of plastic seedling trays creates waste management challenges, with inadequate recycling leading to potential “white pollution.” Additionally, large-scale substrate consumption (particularly of soil and coconut coir) poses sustainability concerns if not properly managed.
4. Practical Application - Standardization Barriers and Integrated Development: The lack of unified seedling-tray specifications (currently three standardized sizes) and interface standards results in poor equipment universality and interoperability. There is a significant mismatch between sophisticated technological solutions and the practical needs of small-scale farmers, creating an adoption paradox. Furthermore, the “last-mile” challenge of transporting seedlings from centralized nurseries to dispersed fields adds substantial logistical complexity and cost.

### 5.2 Future development trends

1. Integration of Intelligent Logistics System: An intelligent logistics system combining AGVs (Automated Guided Vehicles) and track matrices is adopted to enable the full-process autonomous transfer of seedling trays from the sowing area to the hardening area and transplanting area. For instance, after a seedling nursery base in Shouguang, Shandong Province applied this technology, the transfer efficiency of seedling trays increased from 2,000 trays per day (manual operation) to 8,000 trays per hour, and the seedling damage rate dropped from 15% to below 3%. The system dynamically plans the optimal path through the WMS (Warehouse Management System), achieving seamless connection with equipment such as seeders and transplanters with an error of less than 5 mm. Additionally, it supports the expansion of multi-layered stereoscopic shelves, increasing space utilization to 85%.
2. Service-Oriented Promotion: Establish Agricultural Service Centers that promote industrialized seedling cultivation combined with unified agricultural supplies procurement and full-process managed services. To provide factory seedling service and mechanized transplanting service for small farmers in the surrounding area, which can not only recover the mechanical cost but also drive small farmers to complete the whole process of rice mechanization. Simultaneously achieving rapid cost recovery for agricultural service centers and increased income and yield for surrounding small-scale farmers. This model can reduce material and labor costs by ¥80–200 per mu for smallholders while ensuring seedling quality and planting efficiency. Multifunctional application: It can be used to raise vegetables and other crops during the idle time, and increase the utilization rate of the seedling factory.
3. Eco-friendly Materials: Develop and promote biodegradable seedling trays and anti-lodging substrates suitable for mechanical operations. Standardized Design of Seedling Trays: The promotion of universal seedling trays conforming to the LY/T 1720–2007 standard requires standardized tray dimensions (e.g., 480mm × 320mm) and structures. This ensures compatibility with transplanters, logistics equipment, and other related machinery, laying a foundation for the smooth connection of the entire mechanized workflow. Renewable Energy Integration: Incorporate solar power systems to offset electricity consumption in seedling greenhouses. Circular Agricultural Practices: Establish substrate recycling systems and explore local, sustainable alternative materials to reduce resource extraction.
4. Enhanced Agricultural Integration: Develop supporting agricultural materials such as dwarfing and strong-seedling agents, establishing an integrated technical system of “appropriate machinery + optimized methods.” Unified Standards Development: Accelerate the establishment of industry-wide standards for seedling trays and equipment interfaces to improve compatibility and reduce costs. Adaptive Technology Design: Focus on developing flexible solutions that can accommodate varying farm scales and conditions, particularly through the agricultural service center model that bridges the technological divide for smallholders.
